# Dual x-ray absorptiometry monitoring in pediatric short bowel syndrome: an integrative review

**DOI:** 10.1590/1984-0462/2024/42/2023064

**Published:** 2023-12-22

**Authors:** Angelica Godoi Romagnoli Blum, Tais Daiene Hortencio Russo, Roberto José Negrão Nogueira

**Affiliations:** a Universidade Estadual de Campinas – Campinas, SP, Brazil.

**Keywords:** Intestinal failure, Pediatric, Parenteral nutrition, Absorptiometry, photon, Insuficiência intestinal, Pediatrico, Nutrição parenteral, Absorciometria de fóton

## Abstract

**Objective:**

To analyze the bone health of pediatric patients with short bowel syndrome intestinal failure (SBS-IF).

**Data source:**

An integrative literature review was performed using the data published in the MEDLINE-PubMed and Scientific Electronic Library Online (SciELO) databases between January 2010 and April 2021, and through a manual search of the reference lists of relevant studies. Studies were included if they assessed bone mineral density by the Dual X-Ray Absorptiometry (DXA) technique, incorporated pediatric patients (up to 20 years of age) with SBS under parenteral nutrition (PN) and were written in English. Eleven primary sources met the inclusion criteria for this study.

**Data synthesis:**

Pediatric patients with SBS-IF under long-term parenteral nutrition experienced frequent changes in bone metabolism, leading to osteoporotic fractures and growth failure. These patients have deficiencies in multiple nutrients, such as calcium, magnesium, phosphorus, and vitamin D. Consequently, there are variations in the secretion and regulation of the parathyroid hormone. In addition, the pharmacotechnical limitations related to calcium and phosphorus in the PN solution, use of glucocorticoids, and difficulty performing physical activity are risk factors for the development of metabolic bone disease in pediatric patients with SBS-IF.

**Conclusions:**

Low bone mineral density was associated with a high risk of developing osteoporosis, fractures, and growth deficiency in pediatric patients with SBS-IF on PN therapy in the long term.

## Abstract (pt)

**Objetivo:**

Analisar a saúde óssea de pacientes pediátricos com síndrome do intestino curto — falência intestinal (SIC-FI).

**Fontes de dados:**

Revisão integrativa da literatura usando os dados publicados nas bases de dados Medical Literature Analysis and Retrieval System Online/ United States National Library of Medicine (MEDLINE/PubMed) e Scientific Electronic Library Online (SciELO) entre janeiro de 2010 e abril de 2021 e por meio de busca manual nas listas de referências de estudos relevantes. Foram incluídos estudos em inglês que avaliaram a densidade mineral óssea pela técnica de absorciometria de raio X duplo (DXA), incluíram pacientes pediátricos (até 20 anos de idade) com SIC sob terpia nutricional parenteral. Onze fontes primárias preencheram os critérios de inclusão para este estudo.

**Síntese dos dados:**

A pesquisa revelou que pacientes pediátricos com SIC-FI sob nutrição parenteral (NP) de longo prazo tiveram alterações frequentes no metabolismo ósseo, levando a fraturas osteoporóticas e falha de crescimento. Esses pacientes apresentam deficiências de múltiplos nutrientes, como cálcio, magnésio, fósforo e vitamina D. Consequentemente, houve variações na secreção e regulação do hormônio da paratireoide. Além disso, as limitações farmacotécnicas relacionadas ao cálcio e fósforo na solução de NP, o uso de glicocorticoides e dificuldade para realizar atividade física são fatores de risco para o desenvolvimento de doença óssea metabólica em pacientes pediátricos com SIC-FI.

**Conclusões:**

A baixa densidade mineral óssea foi associada a um alto risco de desenvolver osteoporose, fraturas e deficiência de crescimento em pacientes pediátricos com SIC-FI sob terapia nutricional parenteral em longo prazo.

## INTRODUCTION

According to the guidelines of the European Society for Clinical Nutrition and Metabolism,^1^ intestinal failure is characterized by the inability of the intestine to absorb the minimum amount of water, electrolytes, macronutrients, and micronutrients required to maintain health, nutritional status, and/or growth.

The leading cause of intestinal failure is the short bowel syndrome (SBS), a rare gastrointestinal condition resulting from a significant reduction in intestinal absorption capacity, as a consequence of surgical resection of large portions of the intestine. Resections larger than 50% of the intestine can lead to short bowel syndrome-intestinal failure (SBS-IF).^2,3^ The malabsorption that defines SBS-IF occurs due to the loss of regions essential for the absorption of specific nutrients from the intestinal tract, resulting in the inadequate absorption of calcium, magnesium, and phosphorus and the alteration of mineral homeostasis, contributing to a decrease in bone mineralization.^4,5^

Patients with SBS-IF require parenteral nutrition therapy (PNT) to remain nourished and healthy,^1^ and have a significantly increased risk of developing metabolic bone diseases.^6^ This complication may initially be asymptomatic or manifest as bone pains and pathological fractures.^5,7,8^ There are variations in the concentrations of parathyroid hormone (PTH), toxicity, and vitamin D deficiency in children who receive PNT for a long time.^9,10^ In addition, pharmacotechnical limitations prevent the administration of ideal amounts of calcium, phosphorus, and magnesium, leading to bone demineralization.^9^ Diseases that cause intestinal failure may be associated with metabolic bone diseases. When used as steroids, some drugs can contribute to poor bone formation and increased bone resorption.^11,12^ Low bone mineral content has been described in children with short bowel syndrome during and after weaning off PN.^13–15^

With the best clinical and surgical management of patients with SBS-IF, there has been an increase in their longevity, even though it is often associated with prolonged PNT dependence.^16^ Thus, understanding the factors related to low bone mineral density in pediatric patients with SBS-IF is important, and it allows for timely intervention.

The purpose of this study was to conduct an integrative review of the literature to evaluate the bone health of pediatric patients with SBS-IF using DXA. This research aims to contribute to the knowledge base of pediatric multidisciplinary teams (physicians, nurses, pharmacists, and dietitians), helping understand the factors associated with bone incorporation in pediatric patients with SBS-IF to provide these patients with more appropriate treatment.

## METHOD

To prepare this integrative literature review, the research was guided by the following question: What factors are involved in the bone health of pediatric patients with SBS-IF under long-term PNT?

The integrative literature review was conducted using Whittemore and Knafl’s^17^ method, following the steps of problem identification, searching the literature, data analysis, and presenting the results. The literature review included quantitative literature as per the eligibility criteria, which included an assessment of bone mineral density using the DXA technique. This design was found to be most appropriate for the purpose of this research. The Preferred Reporting Items for a Systematic Reviews and Meta-Analyses (PRISMA) statement^18^ was used to identify, assess, and synthesize the selected studies.

**Figure 1:**
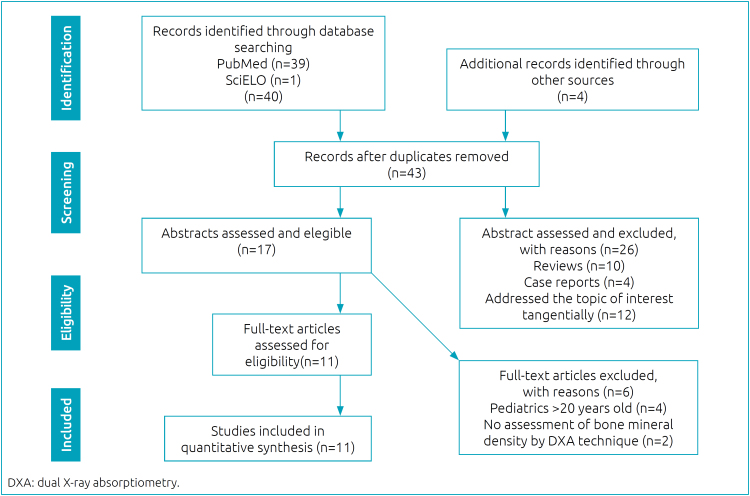
PRISMA flow chart for selection of articles.

According to Whittemore and Knafl,^17^ the following steps were taken: first, we prepared our research questions, and second, we conducted a systematic search of the indexed databases SciELO and MEDLINE-PubMed. The descriptors and their components were based on MeSH (Medical Subject Headings) terms “short bowel syndrome,” “intestinal failure,” and crossed using the Boolean operator “AND” with: “metabolic bone disease,” “bone health,” “bone mineral densitometry,” “bone mineral density,” “bone disease,” “bone deficits,” “growth failure,” “vitamin D deficiency.” To complement the search process, additional articles were sought by manually searching the reference lists of relevant studies. The study identification and selection process, along with the exclusion criteria, is shown in the PRISMA flow chart^18^ (Figure 1).

Searches were performed for the presence of descriptors in the titles and/or abstracts of articles published between January 2010 and April 2021. To be included in this review, articles must have been written in the English language, must have included an assessment of bone mineral density by examining bone densitometry using the DXA technique, and must have incorporated pediatric patients (up to 20 years of age) with SBS under PNT. Duplicate articles, reviews, case reports, and articles that only addressed the topic of interest tangentially were excluded.

A database search identified 44 articles published in English. Two authors screened and read the articles based on the eligibility criteria. After removing duplicates, 43 abstracts were assessed, and 26 were excluded. 17 articles were reviewed and assessed for eligibility. Six full-text articles were excluded as they did not meet the inclusion criteria. The remaining 11 quantitative articles that met the inclusion criteria were included in this integrative review. Figure 1 summarizes the article selection process.

All articles eligible for this review were quantitative, according to the research methodology. Bowling´s^19^ checklist was used to appraise the results. The quality appraisal results using Bowling´s checklist are presented in Table 1. No articles were excluded from this integrative review based on quality scores, as the literature was limited to this subject (bone health in pediatric SBS-IF), and each study´s findings were congruous with the themes.

**Table 1:** Quantitative studies critical appraisal checklist.

	Criteria	Appleman et al.^20^	Demehri et al.^16^	Diamanti et al.^13^	Khan et al.^24^	Kvammen et al.^25^	Louazon et al.^21^	Mutanen et al.^22^	Nader et al.^23^	Olieman et al.^14^	Pichler et al.^12^	Poinsot et al.^9^
1	Aims and objectives clearly stated	+		+	+	+	+	+	+	+	+	+
2	Hypothesis/research questions clearly specified	+	+	+	+	+	+	+	+	+	+	+
3	Dependent and independent variables clearly stated	+	+	+	+	+	+	+	+	+	+	+
4	Variables operationalized	+	+			+	+		+	+		+
5	Design adequately described		+	+	+	+	+	+	+	+	+	+
6	Appropriate methods	+	+	+	+	+	+	+	+		+	+
7	Instruments used tested for reliability and validity	+	+	+	+	+	+	+	+	+	+	+
8	Source of sample, inclusion/exclusion, and response rates described	+	+	+	+	+	+	+	+	+	+	+
9	Statistical errors discussed									+		+
10	Ethical considerations	+		+	+	+		+	+	+	+	+
11	Was the piloted study						+					
12	Statistically appropriate analysis	+	+	+	+	+	+	+	+		+	+
13	Results reported and clear	+	+	+	+	+	+	+	+	+	+	+
14	Results reported related to hypothesis and literature	+	+	+	+	+	+	+	+	+	+	+
15	Limitations reported	+	+	+	+	+	+	+		+		+
16	Conclusions do not go beyond limit of data and results		+				+			+		
17	Findings able to be generalized								+			
18	Implications discussed	+	+	+	+	+	+	+	+	+	+	+
19	Existing conflict of interest with sponsor					+						
20	Data available for scrutiny and reanalysis	+	+	+	+	+	+	+	+	+	+	+

The articles included were then analyzed by extracting, organizing, and reducing the data to individual tables. The data were synthesized based on the following criteria: authors, year of publication, country where the study was conducted, type of study, study period, number of participants and results. The findings are presented in Table 2. Data such as age, bone mineral densitometry results, time of PN and DXA assessments and time after weaned off PN are compiled in Table 3.

**Table 2:** Description of studies included in this review.

Author/Country	Type of study	Study period	N	Results/findings
Appleman et al.^20^/USA	Cross-sectional	Not reported	20	Vitamin D deficiency, growth retardation
Demehri et al.^16^/USA	Retrospective, cohort	2006–2012	36	Fractures, bone pain, vitamin D deficiency*, hyperparathyroidism
Diamanti et al.^13^/Italy	Longitudinal prospective	2005–2007	24	Fractures
Khan et al.^24^/USA	Retrospective, cohort	2004–2013	65	Vitamin D deficiency*, hyperparathyroidism
Kvammen et al.^25^/Norway	Observational cross-sectional	2007 (March to September)	69	Impaired growth
Louazon et al.^21^/France	Cross-sectional, case-control	March 2014 to June 2015	11and 22 (patients and controls)	No diferences for bone densities in patients undergoing chronic PN
Mutanen et al.^22^/Finland	Cross-sectional	1984–2010	41	Fractures, vitamin D deficiency, hyperparathyroidism
Nader et al.^23^/France	Single center retrospective, cross-sectional	January 2016 to December 2018	40	Bone fractures* after a mild trauma, and vitamin D deficiency*
Olieman et al.^14^/Netherlands	Cross-sectional	2005–2007	31	Short stature
Pichler et al.^12^/Austria	Prospective, cohort	2002–2010	12	Short stature
Poinsot et al.^9^/France	Retrospective, cohort	2004–2014	31	Vitamin D deficiency

* No statistical significance.

**Table 3:** Age, Bone Mineral Densitometry results, time of parenteral nutrition, dual X-ray absorptiometry assessments and time after weaned off parenteral nutrition of pediatric patients with short bowel syndrome.

Author	Age	BMD	Time of PN	DXA assessments and time after weaned off PN
Appleman et al.^20^	Median: 26 Min-Max (6–127) months	Lumbar (15%), Total (12%), ↓ in IF than the control group	Median: 18.5 Min-Max (4–103) months	Not reported
Demehri et al.^16^	≥6 years and diagnosed until 18 years	Lumbar: Mean±SD: -1.36±1.47 First DXA Mean±SD: -1.36±1.18 Second DXA	Mean±SD: 5.1±5.4 years	Not reported
Diamanti et al.^13^	Mean±SD: (5.1±2.9) years	Total Mean±SD: -2.5±1.2*	Mean±SD: 32.4±28.8 months	Not reported
Khan et al.^24^	≥5 to 20 years	DXA less or equal–2 Mean±SD: 1772.9±1453.7 DXA greater–2 Mean±SD: 1126.1±1194.3 p=0.09 ↓ 22 childrens (22/65)–40%	Mean±SD: 44.2± 43.2 (unity)	DXA less or equal – 2 Mean±SD: 2127.9±1921.9 DXA greater – 2 Mean±SD: 2187.2±1300.0 p=0.92 32 days
Kvammen et al.^25^	Mean±SD: 10.1±3.5 years	Total median: -0.4 and Lumbar: -0.9	>6 months	Not reported
Louazon et al.^21^	Median: 16 Min-Max (9–19) years	Spine total: Median (Min-Max) and BMC (g): 40 (14–66) and BMD (g/cm²): 0.8 (0.4–0.9) and Whole body: Median (Min-Max) BMC (g): 1198 (441–1832) and BMD (g/cm²): 0.81 (0.54–0.96)	Median: 10.3 Min-Max (6.4–18.3) years	Not reported
Mutanen et al.^22^	Mean: 9.9 Min-Max (0.2–27†) years	Lumbar: Mean: -2.1 Min-Max (-4.1–0.9) Femur: Mean: -0.9 Min-Max (-2.7–1.1)	Mean: 41 Min-Max (0.7–250) months	Lumbar: Mean: -1.2 Min-Max (-3.2–0.9). Femur: Mean: -0.8 Min-Max (-2.6–0.7) 9.0 years min-max (0.3–27)
Nader et al.^23^	Mean±SD: 12.4±4.5 years Median: 12.2 years	BMI mean±SD: -0.5±1.3 – Median: -0.4 BMI ≤-2 SD [n(%)]: 7 (18)	Mean±SD: 12.4±4.4 years Median: 12.4 years	Not reported
Olieman et al.^14^	Mean±SD: 11.8±4.2) years	Total: Mean±SD: -0.04±1.4 and Lumbar: Mean±SD: -0.47±1.2 – BMC: Mean±SD: -1.0±1.1	Median: 110 Min-Max (43–2345) days	Not reported
Pichler et al.^12^	5 to 14 years	Total: Mean±SD: -1.3±1	Median: 4.5 Min-Max (2–11.4) years	Not reported
Poinsot et al.^9^	Median 2.9 Min-Max (0.40–13.3) years	TBMC‡ Median: -1.9(-5.3 to 2.6) – first DXA TBMC‡: Median: -1.14(-2.25 to 1.84) – Last DXA	Median: 2.7 Min-Max (0.1–12.7) years	Not reported

BMD: bone mineral densitometry; PN: parenteral nutrition; DXA: dual X-ray absorptiometry; SD: standard deviation; IF: intestinal failure; BMC: bone mineral content; TBMC: total bone mineral content.

* p<0.001

† adults with infantile SBS

‡ Low bone mass was defined by a TBMC z score ≤-2).

## RESULTS

11 studies published between January 2010 and April 2021 met the inclusion criteria. These studies were conducted in Europe (8) and the United States (3). All studies were quantitative, including retrospective cohort (3), longitudinal prospective (1), prospective cohort (1), and transversal/cross-sectional (6) studies. The follow-up duration varied between 6 months and 26 years. The number of samples in these studies ranged from 11 to 69.

According to the inclusion criteria, Bone Mineral Density (BMD) was assessed using DXA. In these studies, bone density was measured by the following scanners: DXA Hologic Inc. (72.7%), GE Lunar Prodigy (27.3%). 63.6% studies reported low BMD values in the lumbar region, and 81.8% reported low total BMD values.

Among the 10 (90.9%) studies involving evaluation of patients by BMD, fractures were reported in 70% of patients, 28.5% of whom were considered to have pathological fractures. Appleman et al.^20^ reported 5% of fracture in each group (study and control group), but they did not determine whether it was related to risk factors for low bone density. In the study by Demehri et al.,^16^ 11.1% of patients had pathological fractures: humerus and vertebral compression fractures, bilateral femur fractures, multiple vertebral fractures with bilateral fractures of the femur, and bilateral fractures of the radius and ulna. According to Diamanti et al.,^13^ 8.3% of patients with SBS developed non-spinal fractures (forearm and femur fractures). As per Louazon et. al.,^21^ 36.3% of patients and 4.5% of controls had a history of long bone fracture, none had a vertebral fracture, and none had more than two fractures. All were traumatic fractures. Mutanen et al.^22^ reported that 4.8% of patients had persistent peripheral fractures, and that vertebral compression suggesting osteoporotic fracture was present in 4.8% of patients. Nader et al.^23^ reported that 5% of patients in their cohort had a history of trauma-associated fractures. Demehri et al.^16^ reported that 16.7% of patients had bone pain (Table 2).

Olieman et al.^14^ identified that their mean weight for age and height for age were significantly lower than the reference values (p=0.005 and p=0.001, respectively). The mean height for age was significantly lower (p=0.000) than the target height (TH). In total, 53 % were below their TH range. Pichler et al.^12^ reported that the height SDS was less than –2 in 5 (42%) of 12 children with short bowel syndrome, which was defined as growth failure.

Appleman et al.^20^ reported that participants in the intestinal failure group had a mean serum 25(OH)D concentration of 39.5 mg/mL. The authors used the Institute of Medicine as a reference, which specified a limit for serum 25(OH)D concentration of >20 ng/mL (50 nmol/L) as sufficient vitamin D for almost all children; however, the authors acknowledge that there is controversy regarding the optimal serum concentrations of 25(OH)D, and that others have argued for a higher cutoff. In the studies by Demehri et al.^16^ and Khan et al.,^24^ patients had vitamin D deficiency (63.8% and 41%, respectively) and a correlation with low bone mineral density; however, the difference was not statistically significant. The authors argue that this may be due to the very small cohort used in the studies, which would require a larger cohort for corroboration. Demehri et al.^16^ defined vitamin D deficiency as a serum 25(OH)D level <30 ng/mL. Khan et al.^24^ defined vitamin D deficiency as a serum 25(OH)D value of 30 ng/mL. Kvammen et al.^25^ found sufficient levels of 25 (OH)D in both groups: 28.4 ng/mL (71 nmol/L) in the IF group and 32.4 ng/mL (81 nmol/L) in the healthy group. Vitamin D sufficiency was defined as a total serum 25 (OH)D level >20 ng/mL (50 nmol/L). Louazon et al.^21^ defined a partial deficiency could be considered in patients and controls since vitamin D levels should be higher than 30 ng/mL (75 nmol/L). The recommendation for parenteral vitamin D intakes are 400 UI/day. All patients had oral supplementation: 100 000 UI of vitamin D3 every 3 months. Median vitamin D3 intake in PN was 220 (0 min–250 max) UI/day. The biological paramenters of 25 hydroxy-vitamin D were similar between the two groups: 17.6 (9.6 min–48.8 max) ng/mL [44 (24 min–122 max) nmol/L]. Therefore, intestinal malabsorption could explain their low plasmatic level of vitamin D. Mutanen et al.^22^ reported that vitamin D deficiency was found in 44% of patients despite receiving increased amounts of vitamin D in their daily diet and vitamin D supplements. The reference values used were as follows: serum 25(OH)D <15 ng/mL (37.5 nmol/L) was considered vitamin D deficiency; between 15 and 20 ng/mL (37.5 and 50 nmol/L), vitamin D insufficiency; >25 ng/mL(50 nmol/L), vitamin D deficiency; and >32 ng/mL (80 nmol/L), the ideal level. Nader et al.^23^ reported that the mean concentration of 25-hydroxyvitamin D3 (25-OHD3) in their cohort was 26.5 ng/mL (66 nmol/L). In their department, a child is considered to have a sufficient level of 25-OHD3 when it is >30 ng/mL (75 nmol/L). As for the deficiency definition, the cut-off is < 10 ng/mL (25 nmol/L). A low cut-off was chosen because children with increased PTH levels and those suffering from osteomalacia or rickets had 25- OHD3 levels. The normal values of 25-OHD3 were 30–60 ng/mL or 75–150 nmol/L. They supplemented children whose 25-OHD3 level was <20 ng/mL (50 nmol/L). Pichler et al.^12^ found no significant differences in bone mineral density according to the diagnosis, and 25(OH)D deficiency. Reference values for serum 25(OH)D concentration of 25 ng/mL were considered vitamin D insufficiency. In a study by Poinsot et al.,^9^ the mean plasma vitamin D level was low at baseline, 15.4 ng/mL (38.5 nmol/L), and did not increase at the endpoint at 14 ng/mL (35 nmol/L), which probably indicates that intestinal calcium absorption has not been promoted. Reference value: a vitamin D level of 32–100 ng/mL (80–250 nmol/L) was considered a normal value (Table 2).

Appleman et al.^20^ reported median serum PTH values of 51.2 pg/mL and 98.1 pg/mL in FI and control groups, respectively, which can lead to decreased bone renewal and bone mass. The lower PTH level may be the result of the continuous infusion of calcium in the PN required for patients with IF or higher serum aluminum. Demehri et al.^16^ reported secondary hyperparathyroidism in 9/36 patients (25%). A reference value of serum PTH between 10.0 and 55.0 pg/mL was considered normal. Khan et al.^24^ found that PTH serum levels were significantly higher in the IF group; however, multivariable models did not identify this as an independent association, possibly because it is a covariate with low levels of serum calcium and vitamin D. Louazon et al.^21^ identified that patients displayed a relative hyperparathyroidism: PTH levels were within the normal range, but significantly higher in patients as compared to controls (256% of controls; p=0.003). Mutanen et al.^22^ studied secondary hyperparathyroidism in most patients (44%) with vitamin D deficiency. Hyperparathyroidism was absent when serum 25(OH)D level was >80 nmol/L. A reference value of PTH >47 ng/L was considered secondary hyperparathyroidism. Nader et al.^23^ reported that the mean concentration of parathyroid hormone (PTH) was in the normal ranges. Eight children (20%) had PTH levels above normal, with low 25-OHD3 levels. Serum PTH was defined as elevated when >55pg/mL. The mean concentration of PTH level was 39 ng/L.

Demehri et al.^16^ stated that there was a limitation in their study in relation to the assessment of the variation in calcium, phosphorus, and vitamin D content in the parenteral nutrition formulations of individuals. Supplementation with calcium and phosphorus via parenteral nutrition is limited by the precipitation of these minerals.

Pichler et al.^12^ found no significant differences in bone mineral density based on the diagnosis and use of steroids.

In a study conducted by Kvammen et al.^25^ to assess usual physical activity level, the participants completed a questionnaire on the frequency per week of sessions lasting more than 30 min (“never or less than once,” “1 and 3 sessions,” “4 sessions or more”). Participation in different sports and intensity of physical activity were also included in the questionnaire. An overall lower level of physical activity participation was found in patients with IF as compared to healthy subjects. A significant difference in sports participation was detected (26% vs. 2%), and the intensity of physical activity participation was lower in patients with IF than in healthy participants.

In studies by Khan et al.^24^ and Mutanen et al.,^22^ time since weaning of PN was 32 days and 9.0 (0.3–27) years, respectively.

## DISCUSSION

It was found that metabolic bone disease is common, detectable, and causes a considerable burden on the health of patients with SBS-IF under PNT in the long term; therefore, it is important to evaluate it routinely. The investigation for this evaluation is bone densitometry, which uses ionizing radiation to obtain images of bones.^26,27^ All the studies analyzed the risk of metabolic bone disease by using bone densitometry.

Although BMD assessment is usually more commonly adopted after 50 years of age, it is also performed to detect conditions of low bone mineral density in children with chronic diseases.^28^ Another application of BMD is monitoring the effectiveness of therapeutic techniques, such as the use of bisphosphonates in patients with primary bone diseases.^29,30^

The ideal BMD assessment technique for children is dual-energy X-ray absorptiometry (DXA), a two-dimensional imaging technique that uses flat images to estimate the bone area. This is recommended because it is fast, safe, widely available, and accurate, with an effective radiation dose ranging from 0.02 to 0.03 μSV.^31^ DXA uses a low radiation dose that requires the patient to remain immobile during the examination, which is a difficult task for younger children or patients with intellectual or neurological disabilities.^32^

The cause of metabolic bone disease in PNT in the long term appears to be multifactorial, and several hypotheses have been proposed to explain it.^33^ Pediatric patients with SBS-IF exhibit malabsorption of macronutrients, vitamins, water, electrolytes, and minerals, resulting in hydroelectrolytic abnormalities and malnutrition.^34,35^ Metabolic changes due to loss of regulation of the colon, gastric function, and small intestine lead to the depletion of calcium, magnesium, and vitamin D, resulting in bone demineralization and a low BMD.^36^

Mutanen et al.,^22^ Demehri et al.^16^ and Olieman et al.^14^ reported that low BMD has harmful health consequences, including osteoporosis, fractures, and insufficient growth. The fractures presented in these studies were due to osteoporosis. Osteoporosis is a systemic skeletal disorder characterized by low bone mass and micro-architectural deterioration of bone tissue, with a consequent increase in bone fragility and susceptibility to fractures.^37^

According to Mutanen et al.^22^ and Demehri et al.,^16^ children with osteoporosis may have recurrent long-bone fractures, even if associated with low-impact trauma. Spinal compression fractures can still occur with a decreased final height and multiple spinal deformities.^28^ These fractures can be asymptomatic, and can only be identified when spinal radiography is performed in the context of investigating low bone density.^28^

The risk factors for osteoporosis are vitamin D and calcium deficiency, secondary hyperparathyroidism, and the use of glucocorticoid.^28^

Under normal circumstances, vitamin D regulates the synthesis and release of parathyroid hormone (PTH) and stimulates intestinal calcium absorption, causing an increase in serum calcium that is used in bone formation.^38^ However, in children with SBS-IF, due to multiple resection surgeries, the function and adequacy of the length of the small intestine are compromised, directly and negatively interfering with the absorption of calcium and vitamin D.^39,40^ If there is an associated vitamin D deficiency, there will be a decrease in plasma calcium levels and, as a consequence, secondary hyperparathyroidism. This causes an imbalance in osteoblasts, osteoclasts, and bone metabolic diseases.^38^

Vitamin D requires micellar solubilization by bile acids; therefore, absorption is impaired by malabsorption of bile acid after resection.^40^ In addition, inadequate vitamin D concentration is frequent due to low sun exposure and inadequate intake. Furthermore, there is not always enough vitamin D in the PNT to avoid this deficiency.^41^

Vitamin D is typically acquired via exposure to sunlight, particularly UVB radiation.^10^ Children with SBS-IF have many complications due to long-term use of PNT, resulting in frequent hospitalizations with a potential decrease in adequate exposure to sunlight, consequently resulting in inadequate concentrations of vitamin D.^10^ Other factors that can affect vitamin D status include sunscreen use, skin pigmentation, latitude, and season.^10^ A relevant fact is that all the studies presented in this review were carried out in the Northern Hemisphere (United States of America and Europe), with greater latitude variations, more severe winters, and consequently less exposure to sunlight, increasing the possibility of a vitamin D deficiency in children with SBS-IF.

A very important factor is that prolonged PNT, although generally enabling the supply of the necessary macronutrients, does not do so in full in relation to the minerals involved in bone metabolism in these patients.^9^ This is mainly because of the pharmacotechnical limitations involved in the compatibility of this solution.^24^ In fact, Demehri et al.^16^ reported in their work that the infusion of calcium and phosphorus has limitations related to the pharmacotechnics of the PN prescription itself, making the balance of bone mineralization to maintain skeletal mass a challenge.

In addition, patients with SBS-IF require large amounts of calcium, phosphorus, and magnesium.^5,42^ This situation is aggravated when long-term PNT occurs in childhood, because approximately 90% of the peak bone mass is acquired by 18 years of age.^43,44^ This cumulative deleterious effect is related to an increased risk of fractures and osteoporosis.^42^

Pichler et al.^12^ concluded that another factor contributing to metabolic bone disease in patients with SBS-IF is the use of glucocorticoids for the treatment of diseases that may have caused SBS, such as inflammatory diseases. Glucocorticoids affect the function of osteoblasts by reducing their formation and increasing bone resorption. In addition, they reduce intestinal absorption and increase renal tubular calcium excretion.^28^

Louazon et al.^21^ and Nader et al.^23^ excluded patients with intestinal failure who used glucocorticoids from their studies, and concluded that high dependence on PN and very long-term PN (>10 years) do not appear to increase the risk of growth failure or BMD.

Louazon et al.^21^ is a monocentric study with a small cohort, and this study was designed as a pilot study, which may have influenced the reported results.

Physical activity, especially strength and medium-impact exercises, is fundamental for bone health and contributes to maintaining and improving bone mineral density and preventing bone loss.^45,46^ Children with SBS-IF have limitations in practicing physical activities because of the prolonged infusion of PNT and frequent hospitalizations for the treatment of complications related to PNT and underlying disease. Therefore, there is a decrease in the time available for physical activity.^25^ In the present review, only Kvammen et al.^25^ evaluated the physical activity of subjects. A lower frequency physical activity was found in patients with IF as compared to healthy children, which may be an important factor contributing to the reduction in bone mineral density.

Regarding the time of PN use, Mutanen et al.^22^ found that the majority of IF patients show decreased BMD with poor vitamin D status and secondary hyperparathyroidism. These conditions persist not only during the use of PN, but also after the patients have been weaning off PN. Bone changes can still be observed several years following the discontinuation of PN.

Another important factor refers to the thyroid metabolism. In fact, it is known that hypothyroidism can cause resistance to PTH. In the PN solutions there is no addition of iodine, a fundamental mineral for the thyroid. Thus, cases of SBS with hypothyroidism due to iodine deficiency have been described, which alerts us to the monitoring of thyroid-stimulating hormone levels in cases of SBS.^47^

A better understanding of the factors associated with bone incorporation in patients with SBS-IF is the first step toward adequate intervention. It is a fact that children with SBS-IF are exposed to several risk factors for developing mineral bone disease. Therefore, the periodic monitoring of these patients is extremely important. With early intervention, the risk of osteoporosis-related fractures and their impact on growth, quality of life, and longevity are minimized.^43,44^

This integrative review had some limitations. Eligible studies presented heterogeneous results, making it difficult to compare them. Another limitation is that all studies were performed in the Northern Hemisphere, which has more severe winters, with the possibility of patients presenting vitamin D deficiency and consequently low bone mineral density.

In the present review, it was possible to identify risk factors for bone demineralization and emphasize the importance of monitoring bone density using DXA. Low bone mineral density was related to a high risk of developing osteoporosis, fractures, and growth deficiency in pediatric patients with SBS-IF under PNT in the long term.
